# BMRC: A Bitmap-Based Maximum Range Counting Approach for Temporal Data in Sensor Monitoring Networks

**DOI:** 10.3390/s17092051

**Published:** 2017-09-07

**Authors:** Bin Cao, Wangyuan Chen, Ying Shen, Chenyu Hou, Jung Yoon Kim, Lifeng Yu

**Affiliations:** 1 College of Computer Science, Zhejiang University of Technology, Hangzhou 310023, China; bincao@zjut.edu.cn (B.C.); cwy.zjut@gmail.com (W.C.); shenying@zjut.edu.cn (Y.S.); houcy.zjut@gmail.com (C.H.); 2 Graduate School of Game Gachon University 1342 Seongnam Daero, Sujeong-Gu, Seongnam-Si, Gyeonggi-Do 461-701, Korea; 3 Hithink Royal Flush Information Network Co., Ltd., Financial Information Engineering Technology Research Center of Zhejiang Province, Hangzhou 310012, China; yulifeng@myhexin.com

**Keywords:** Internet of Things (IoT), sensor monitoring networks, bitmap, maximum range counting

## Abstract

Due to the rapid development of the Internet of Things (IoT), many feasible deployments of sensor monitoring networks have been made to capture the events in physical world, such as human diseases, weather disasters and traffic accidents, which generate large-scale temporal data. Generally, the certain time interval that results in the highest incidence of a severe event has significance for society. For example, there exists an interval that covers the maximum number of people who have the same unusual symptoms, and knowing this interval can help doctors to locate the reason behind this phenomenon. As far as we know, there is no approach available for solving this problem efficiently. In this paper, we propose the Bitmap-based Maximum Range Counting (BMRC) approach for temporal data generated in sensor monitoring networks. Since sensor nodes can update their temporal data at high frequency, we present a scalable strategy to support the real-time insert and delete operations. The experimental results show that the BMRC outperforms the baseline algorithm in terms of efficiency.

## 1. Introduction

Sensor monitoring networks [1,2] are advanced technology that consist of several sensor nodes. Within sensor monitoring networks, temporal data is steadily collected by deployed sensor nodes while observing physical phenomenon [3]. For example, temporal data from health, climate and earthquakes [4], etc., are usually sensed and transmitted in sensor monitoring networks. Moreover, the specific time interval which records the maximum number of specific events is of great importance in our daily life.

For instance, health-care is an important domain investigated by many people. Postprandial blood glucose [5] has been recognized for a long time as a risk factor for cardiovascular disease, especially the non-insulin-dependent diabetic patients. Generally, diabetic patients will experience blood glucose peaks after a meal, which will increase the risk of cardiovascular disease, if they do not get standard glucose control. However, the time horizon in which the postprandial blood glucose levels of most patients shows a sudden spike is not clear. Hence, the certain time interval during which the maximum number of patients get their blood glucose spike will be of great importance for medical institutions. We can consider this a typical maximum range counting problem. In this case, we are supposed to collect each patient’s temporal period during which postprandial blood glucose spike occurs. For simulation analysis, we can imagine that diabetic patients were embedded by a wearable sensor to record their blood glucose.

However, there exists several challenges which are hard to solve: (1) a single sensor will generate temporal data [6] while sensing the outer environment in real time, which will cause many updating events [7], such as temporal data insertion and deletion operations; (2) sensor monitoring networks must contain thousands of sensors that would produce large-scale temporal data. Generally, the conventional approach tends to traverse the entire temporal data violently, that is to say, using a time window of a given size along a time axis to get the sensor set results of each interval, then we select the optimal interval from them. However, the time consumption of this approach is too huge to process large-scale temporal data efficiently. Besides, it doesn’t support updating temporal data.

In this paper, we present BMRC, a new scalable bitmap-based maximum range counting approach that avoids the drawbacks of the existing techniques. BMRC gets the optimal interval that records the highest incidence of events by processing large-scale temporal sensor data, while taking into account: (a) the time cost of updating temporal data—each sensor in the monitoring network will produce a great deal of temporal data while capturing a certain event, so it is important to support updating affairs in BMRC; (b) the query processing performance, that is to say, BMRC can get the optimal interval by processing the whole temporal data in a short period of time. e.g., during a month, ten thousand diabetic patients are monitored in a sensor monitoring networks, where each diabetic patient is monitored by an embedded sensor [8]. If there is a blood glucose spike over the normal value monitored by the sensor, the occurrence and termination time will be recorded. In this way, every diabetic patient will be persistently monitored for a month. After that, BMRC will process the temporal sensor data and encode it to a bitmap index structure [9] for query processing. Finally, based on the bitmap index structure and a given time window, we can query the optimal interval which results in the maximum number of diabetic patients suffering hyperglycemia.

The results of simulation experiments show the scalability and good performance of BMRC. BMRC can get the optimal time interval in a few seconds by processing tens of thousands of temporal sensor data in real time. Moreover, the BMRC approach supports the insert and delete operations of temporal sensor data in few seconds, which can meet the needs for frequent temporal data updating events in sensor monitoring networks. In general, the contributions described in this paper can be summarized as follows:

1. We define the maximum range counting problem that can satisfy the requirement of finding the optimal interval that result in the maximum occurrence number of the same event monitored by each sensor in a sensor monitoring network.
2. We propose BMRC that can solve the above problem by making use of all temporal sensor data generated in the sensor monitoring network. The proposed approach can rapidly respond to the query requests from outside. Moreover, the insert and delete operations of temporal sensor data are well supported in BMRC.
3. Extensive experimental evidence has demonstrated the high scalability and efficiency of our proposed approach.

We begin the paper in Section 2 by describing the related work, while in Section 3 we introduce the preliminary notions of the approach. Then we introduce the bitmap index construction procedure for the following query approach in Section 4. Section 5 presents the underlying approach for the following sections. Then, in Section 6, we conduct experiments on simulation of temporal sensor data and present the results. Section 7 concludes this paper.

## 2. Related Work

In recent years, sensor monitoring networks have been frequently deployed to observe and record objects in motion, the climate and human health. Typical sensor monitoring networks may consist of tens of thousands of sensors, embedded in different physical spaces, persistently collecting and transmitting their temporal data to a database. Existing research on temporal sensor data processing can be summarized in several categories: TAG [10,11] is a tiny aggregation service for ad-hoc sensor networks. Zhao et al. [12] described an architecture for computing aggregates for monitoring wireless sensor networks. The above two approaches share the same technique of sensor data processing. However, their general research objective is distributed computation of aggregate queries, while BMRC pays more attention to the maximum number of sensors who can cover the same optimal interval, which is different from the above approaches. Yin et al. [13] proposed prediction systems that can provide query services in sensor monitoring networks. They aimed at providing locational query services for users. Moreover, this query service can be distributed and processed in the sensor network, however, any sudden change of temporal data detected in these architectures will consequently cause changes in more details, which will result in huge time consumption.

Sgroi et al. [14] suggested a set of services and an API to free developers from the details of sensor networks. However, their focus is on the systematic definition and classification of sensor data, while BMRC takes a more particular view and provides a management structure for a certain area. Hourglass [15] provides an Internet-based infrastructure for connecting sensor networks [16] to applications and offers topic-based discovery and data-processing services. Like BMRC it tries to hide the internals of sensors from the users but focuses on maintaining quality of query service under the condition of disconnections, while BMRC is more targeted at flexible sensor data updating and query support.

The maximum range counting problem is also different from the conventional temporal aggregation query problems such as COUNT, MAX, MIN and AVERAGE which focus on querying for a certain point time [17]. None of the above approaches can solve the maximum range counting problem, which distinguishes BMRC from them. Moreover, the maximum range counting problem can also be applied to areas other than sensor network, e.g., service [18] and mobile computing [19], hence, it is meaningful to solve this problem.

## 3. Preliminary Notions

This section presents a set of preliminaries that are important to set the stage for understanding BMRC and its vision. In particular, we discuss our research object in BMRC by defining the concepts of data object, the concept of time window, the problem definition, and the underlying data structure.

### 3.1. Data Object

A certain monitoring sensor generates several temporal data every day, where each piece of the temporal data describes the information of a monitored event. For example, the blood glucose of a diabetic patient monitored above the standard value in the time between 11:20–11:30. Let us consider the time interval (11:20, 11:30) as a piece of temporal data. Hence, our research object is a series of temporal data generated by all sensors in sensor monitoring networks, which contains two elements: (1) *SensorID—*the identity name of a certain sensor; (2) *Interval*, which denotes a continuous time period during which events occurrence is captured by a monitoring sensor. In particular, *Interval* consists of two variables, *start*, the start time of a certain event, and *end,* the end time of this event. Moreover, each sensor in the sensor monitoring networks may have several *Intervals* during the monitoring period of time. Therefore, there exists a one-to-many relationship between *SensorID* and *Intervals*.

### 3.2. Time Window

The time window represents a time constraint supposed by a certain query request. Specifically, the time window describes the duration of a continuously monitored event, e.g., we need to query the number of diabetic patients whose continuous blood glucose spike time lasts over 1 h. Then we should set a time window to 1 h and query every piece of sensor data to find out the optimal interval that can cover the most patients. Namely, the time window is the prescribed minimum value of all temporal sensor *Intervals*. In other words, before processing temporal sensor data using BMRC, it is necessary to filter out the *Intervals* whose span are less than the duration of the time window. Hence, the time window is one of the important factors that we should focus to evaluate the performance of BMRC. For extensive research, we set up different time windows to evaluate the performance of BMRC, such as 1 min, 5 min, 7 min, etc.

### 3.3. Problem Definition

**Figure 1:**
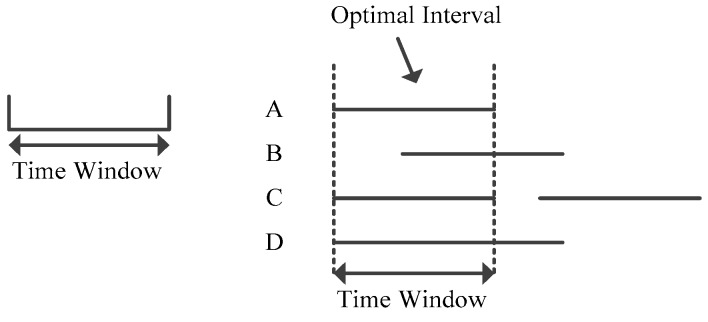
Problem Definition.

Then, based on the understanding of *Data Object*, along with the *Time Window*, we introduce the maximum range counting problem as follows, the whole procedure being as described in Figure 1:

**Definition** **1.** Coverage: the coverage represents the relationship between two time intervals. Besides, it can also describe the relationship between a certain sensor and a certain time interval. For example, when we say an Interval is within the Coverage of another Interval, it means one Interval is the subset of another certain Interval. For two certain Intervals IntervalA and IntervalB, if they satisfy IntervalA ⊆ IntervalB, we can consider that IntervalA is within the Coverage of IntervalB. For another, if a certain sensor has an Interval IntervalA that can cover IntervalB, we consider IntervalB is within the Coverage of this sensor.

**Definition** **2.** *Given a set of sensors S in sensor monitoring networks that monitoring the same event in several different physical spaces, and the corresponding temporal Intervals of each sensor, a given Time Window, BMRC aims to query for the Optimal Interval optI, while meeting the following constraints:*

*1.*  Time Constraint: |optI| ≥ |Time Window|;
*2.*  Coverage Constraints: optI must have the coverage of maximum number of sensors.

### 3.4. Data Structure

BMRC maintains one big table, called *Sensor Table*, which includes an entry for each piece of temporal sensor data currently generated in the sensor monitoring network. Once a certain event occurs, the monitoring sensor *s* will capture the current start time and end time of the event and a new entry for *s* is added to the *Sensor Table* with the following information: a sensor entry *s* has four attributes: (1) *SensorID*: A unique sensor identifier of *s*; (2) *BitPosition*: A number marked by BMRC, start from 0 to n which can be used in bitmap index structure; (3) *Index*: Initialized to 0 which can be used in the procedure of bitmap index structure construction; (4) *Intervals*: A list of temporal *Intervals* generated by its corresponding sensor. Therefore, there exists a one-to-many matching relationship between each *sensorID* in *Sensor Table* and its corresponding *Intervals.*

## 4. Index Construction and Dynamic Updating

### 4.1. Bitmap Index Construction

Based on the data structure *Sensor Table*, this section discusses the details for bitmap construction. The input to this phase is all unique time in *Sensor Table*, the set of sensors stored in the *Sensor Table*. The output is the *EncodedMap* that stores each unique time and its corresponding encoded bitmap.

#### 4.1.1. Main Idea

The target of this phase is to encode a unique bitmap for every unique time which can facilitate the maximum range counting query process. Every sensor’s *BitPosition* denotes if this sensor has an *Interval* that can cover this time. If so, the bit in this *BitPosition* will be marked to TRUE, otherwise it will be marked to FALSE. However, because of the huge number of sensors in *Sensor Table,* the raw encoded bitmap will occupy a lot of storage space in the disk [20], which will also slow down the query time in the following query phase. Moreover, there always appears the same bit in many consecutive data elements in the bitmap index structure. We adopt a Run-Length encoding strategy to compress each bitmap.

#### 4.1.2. Algorithm

Algorithm 1 below presents the pseudo code for this phase. Firstly, we obtain each sensor’s information which includes its *SensorID*, *BitPosition*, *Index* and *Intervals*. Then we scan each time in the *Sensor Table*. For each time in each loop, we process each sensor in *Sensor Table*. For the sensor in each loop, initialize an index value to the sensor’s *Index*. For each i from index to the sensor *Intervals*’ size, initialize a value *I* to the ith *Interval* of the sensor. If *I* contain t, set *BitPosition* to be TRUE in its bitmap, update the *Index* of this sensor to i and break current loop. Otherwise, index auto-increments. If the start time of I is larger than t, break the current loop. Compress the bitmap into a text file using Run-Length Encoding strategy. Read each t and its compressed bitmap [21] from text file and store them into EncodedMap.
**Algorithm 1:****Bitmap Index Construction****Input:**Sensor Table**Output:**A Map Storing Each Unique Time and Its Corresponding Bitmap1.**for** each *t* ∈ *allTimeList*2.  **for** each *s* ∈ *Sensor Table*3.    *index* ← *s*.*Index*4.    **for**
*i* ← *index* to *s.Intervals*. size5.      *I* ← get the *ith Interval* of *s*6.      **if**
*I* cover *t*7.        *bitmap*.set(*s.BitPosition*, true)8.      *s.Index* ← *i*9.        **break**10.      **else**11.        *index++*12.        **if**
*I*. start > *t*13.          **break**14.  *Compressing the bitmap*15.*EncodedMap* ← store each *t* and its compressed *bitmap*

#### 4.1.3. Example

**Figure 2:**
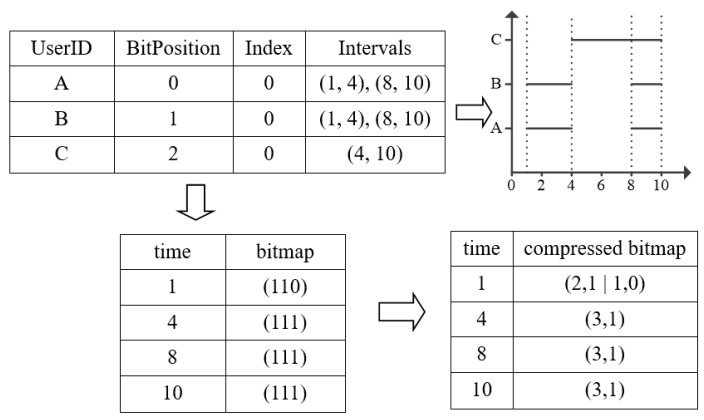
Bitmap Index Construction Example.

Figure 2 gives the *Sensor Table* after the bitmap index construction. Scan each start time and end time in Sensor Table, we will get the *allTimeList* that stores each unique time in ascending order. Then, for every time from 1 to 10 in *allTimeList,* we will scan each sensor from A to C, judge if there exists a Interval of each sensor that can cover this time. e.g., time 8 can be covered by *Interval* (8, 10) in A and B, and *Interval* (4, 10) in C, so the bitmap of time 8 will be set to {111}. In the process of encoding, we proposed a pruning strategy to reduce the time consumption of encoding, the key technique is the Index of each sensor. e.g., when we have scanned the sensor’s ith Interval that cover the time, we will set the Index of the sensor to i to facilitate next time encoding procedure.

**Figure 3:**
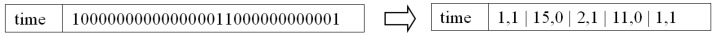
Compressing Bitmap Strategy.

After encoding the bitmap of each time, we will get each time and its corresponding raw bitmap. However, there exist two problems left to be solved. First, since massive numbers of sensor nodes are involved, the raw generated bitmaps (Figure 3) will occupy lots of disk space when they are output to a text file. Second, the traverse of *BitPosition* for calculating sensors’ number in query phase will be time-consuming. Thus, we utilize a Run-Length Encoding strategy [22] to solve the above two problems by compressing each time’s bitmap. For example, as shown in Figure 3, we have a raw bitmap that contains 30 bits in the left part of Figure 3, where the same bit value “0” occurs for 15 times consecutively from the 2nd position. Then, we use the form of “15, 0” to represent this continuous segment within the bitmap. With this, we can derive a corresponding compressed unit as shown in the right part of Figure 3. Each compression unit contains 2 elements: (1) the count of consecutive bit value; (2) the bit value that occurs consecutively. Then we will store the compressed bitmap structure to a text file in the external disk. Finally, each time with its corresponding compressed bitmap will be stored in *EncodedMap* for the query processing and other operations, e.g., insert and delete.

### 4.2. Insert and Delete Operations

After bitmap index structure construction, we get the compressed bitmap index structure. As the sensor monitoring networks always have the requirements to insert and delete temporal data in high frequency. To deal with this situation, we have supposed a scalable strategy to support these updating affairs in the bitmap index structure.

#### 4.2.1. Case 1: Insertion Operation

The algorithm of the insertion operation is described in Algorithm 2. The input in this case is the set of monitoring sensors in *Sensor Table* and a piece of temporal sensor data we want to insert, while the output is the updated *Sensor Table* and *EncodedMap*. First of all, traverse all sensors in *Sensor Table*, make a judgment if the insert Interval’s *SensorID* is exist in the *Sensor Table*. If so, insert the Interval *insertI* into the sensor’s *Intervals* list. Otherwise, the sensorID of *insertI* is new, so we should insert the new sensor’s temporal data into the *Sensor Table*. Because the *BitPosition* of some time’s bitmap may have been changed. Later, we should traverse all the valid time in *Sensor Table*, judging if there exists a *t* that is within *(I.start, I.end)*. If so, re-encode its bitmap in *EncodedMap*.
**Algorithm 2:****Interval Insertion****Input:**A Piece of Temporal Data: *(SensorID, InsertI)* *Sensor Table* *EncodedMap*, generated in *Algorithm 1***Output:**After Insert Processing: *Sensor Table and EncodedMap*1.**if**
*Sensor Table* exists the inserted *sensorID*2.  insert the Interval *insertI* into the sensor *Intervals*3.  **for** each *t* ∈ *Sensor Table*4.    **if**
*t* ∈ *(insertI.start, insertI.end)*5.      set the bit in *BitPosition* of its bitmap into TRUE6.**else**7.  insert this new sensor into *Sensor Table*8.  **for** each *t* ∈ *Sensor Table*9.    **if**
*t* ∈ *(insertI.start, insertI.end)*10.      add a TRUE bit in its bitmap *BitPosition*11.    **else**12.      add a FALSE bit in its bitmap *BitPosition*

#### 4.2.2. Example

**Figure 4:**
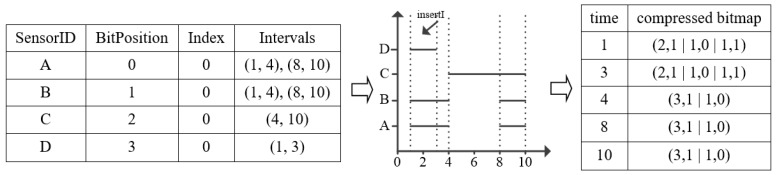
Interval Insertion Example.

Figure 4 shows an insertion example for inserting a new interval whose *SensorID* is D, and the insert *Interval* is (1, 3) into the *Sensor Table* and the *EncodedMap*. Firstly, we should make a judgement if its *SensorID* exists in *Sensor Table*. Obviously, sensor D does not exist in *Sensor Table*, so we insert the information of sensor D into the *Sensor Table*. Then we add a BitPosition of 3 for the new sensor D. For the time in Sensor Table, add a TRUE bit in their bitmap index structure of the time that is within the range of the *(insertI.start, insertI.end)*, while for the time that is beyond the range, and add a FALSE bit in their bitmap index structure. The whole process of insertion is an updating procedure of data structure in *Sensor Table* and *EncodedMap*.

#### 4.2.3. Case 2: Deletion Operation

Algorithm 3 gives the pseudo code for the deletion operation. The input in this case is the set of monitoring sensors in *Sensor Table* and a piece of temporal sensor data we want to delete, while the output is the updated *Sensor Table* and *EncodedMap*. First of all, traverse all sensors in *Sensor Table*, make a judgment if the delete Interval *deleteI*’s *SensorID* is exist in the *Sensor Table*. If so, delete the interval *deleteI* from the sensor’s *Intervals* list. After that, we make another judgment if sensor has no *Interval* after deletion. If so, delete the sensor from the *Sensor Table*. Since the *BitPosition* of some time’s bitmap may have been changed. Later, we should traverse all time in *Sensor Table*, judging if there exists a time that is within *(deleteI.start, deleteI.end)*. If so, encode its bitmap in *EncodedMap*.
**Algorithm 3:****Interval Deletion****Input:**A Piece of Temporal Data: *(SensorID, DeleteI)* *Sensor Table* *EncodedMap*, generated in *Algorithm 1***Output:**After Delete Processing: *Sensor Table and EncodedMap*1.Find the corresponding sensor by the *sensorID*2.Delete the Interval deleteI from sensor Intervals3.**If** the sensor Intervals is empty after deletion4.  Remove the information of the sensor from Sensor Table5.  **for** each *t* ∈ *Sensor Table*6.    Delete the bit in its bitmap *BitPosition*7.**else**8.  **for** each *t* ∈ *Sensor Table*9.    **if**
*t* ∈ *(deleteI.start, deleteI.end)*10.      set a FALSE bit in its bitmap *BitPosition*

#### 4.2.4. Example

**Figure 5:**
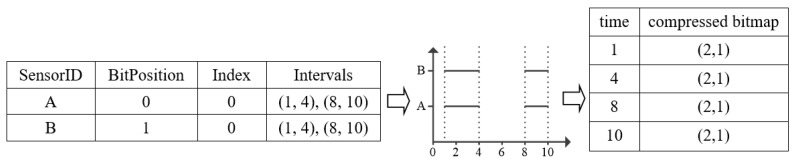
Interval Deletion Example.

Figure 5 gives a deletion example for deleting a temporal data whose *SensorID* is C and the insert *Interval* is (4, 10) from the *Sensor Table* and the *EncodedMap*. Firstly, we should find the corresponding sensor by the delete *sensorID*. Then, delete the *Interval deleteI* (4, 10) from sensor Intervals. After that, we should judge whether the sensor *Intervals* is empty after deletion. If so, remove the entry of the sensor from *Sensor Table*. Then, scan each t in *Sensor Table*, delete the bit in its bitmap *BitPosition* of this sensor. Otherwise, for each t in *Sensor Table*, if t is within the range of *(deleteI.start, deleteI.end)*, set a FALSE bit in its bitmap *BitPosition*.

## 5. Maximum Range Counting

The naive algorithm to support maximum range counting is sliding the time window along the timeline. Meanwhile, during the time window sliding procedure, the time window will move to a new position by a step length which represents the minimum time unit, like 1 s, 1 min and 1 h, etc. Then we are supposed to compute the coverage relation between the time window and the *Interval* of each sensor in *Sensor Table*. If the time window is within the coverage of a certain sensor, we will add it to the alternative sensor set. After that, we will get an alternative sensor set, in which sensors all have an *Interval* that can cover the time window. Through the whole traversal, we will get the largest sensor set with its corresponding optimal interval. Then we merge these optimal intervals if necessary and find out the optimal interval whose sensor set has the largest sensor number. Though the whole algorithm process seems to be simple and easy to implement. The huge time consumption of this approach which takes dozens of minutes to process tens of thousands of temporal sensor data is unacceptable for the maximum range counting query request.

BMRC can obviously reduce the time consumption of query request by encoding [23] all temporal sensor data to a bitmap index structure. BMRC consists of three main phases as follows: (1) *Get Maximum Sensor Set*; (2) *Filter Fake Sensor*; (3) *Adjust the Candidate Interval.* These phases will be described in details in the following sections. For convenient illustration, we use examples throughout the whole sections.

### 5.1. Phase I: Get Maximum Sensor Set

The input of this phase is the map of compressed and encoded bitmap, generated from bitmap construction, the set of sensors stored in the *Sensor Table* and the given duration of time window. While the output is the sensor set that consists of sensors whose *BitPosition* of its bitmap is TRUE after a compressing bit-wise AND [24] operation.

#### 5.1.1. Main Idea

The main idea of this phase is using a compressing bit-wise AND operation to compute the start and end time result in candidate intervals and their corresponding bitmap which can be found in *EncodedMap*. Then we traverse the result bitmap after the compressing bit-wise AND operation and add the sensor whose *BitPosition* is TRUE to a sensor set. The main reason behind this is that if there exists a sensor that can cover the candidate interval, then the bit-wise AND result in the sensor’s *BitPosition* of the candidate interval’s start time and end time’s bitmap will be TRUE.

#### 5.1.2. Algorithm

Algorithm 4 describes the procedure of this phase. Firstly, we should get the corresponding bitmap of start and end time in *EncodedMap,* put them to *BitmapA and BitmapB*. Then initialize a variable *TempBitmap* to store the intermediate result of bit-wise AND operation. After that, we begin to compute each unit in *BitmapA* and *BitmapB* using the compressing bit-wise AND operation and store the result to *TempBitmap*. Moreover, we still need to re-compress the *TempBitmap* into *ResultBitmap*. Moreover, for each bit in *ResultBitmap* in the loop, make a judgement if the bit is TRUE. If so, add the corresponding *BitPosition* sensor into the sensor set.
**Algorithm 4:****Phase I: Get Maximum Sensor Set.****Input:**Start Time and End Time of Candidate Interval. EncodedMap Generated**Output:**The Sensor Set After Bitwise AND Operation1.*BitmapA←start* time’s corresponding bitmap index in EncodedMap2.*BitmapB←end* time’s corresponding bitmap index in EncodedMap3.Initialize a TempBitmap4.  Compute each unit in *BitmapA* and *BitmapB*5.  Add each result unit after computation to TempBitmap6.*ResultBitmap* ← recompress the TempBitmap7.**for** each bit in *ResultBitmap*8.  **if** bit is TRUE9.    Add the corresponding BitPosition sensor to sensor set10.return the sensor set

#### 5.1.3. Example

**Figure 6:**
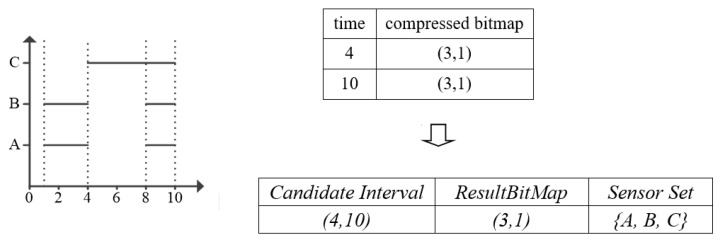
Example for Phase I.

Figure 6 describes a neat example for this phase. The figure gives the part of *EncodedMap* which contains two times 4, 10 with the corresponding compressed bitmap. For the candidate interval [4,10] we compute the two bitmaps of its start time and end time by the compressing bitwise AND operation. After that, we can get the result bitmap (3, 1). Traverse each bit in the bitmap, and add each sensor in the *BitPosition* whose bit is TRUE.

### 5.2. Phase II: Filter Fake Sensor

The input to this phase is the sensor set produced in *Phase I*. The output is the set of sensors after filtering the fake sensors who can’t cover the candidate interval.

#### 5.2.1. Main Idea

The main idea of this phase is filtering out the fake sensors that do not have an interval that can cover the candidate interval completely. Since the result bitmap is generated by the bit-wise AND operation of the corresponding bitmap of start time and end time in candidate interval. The sensors may not have an interval that can cover candidate interval. In order to filter them out from sensor set. We proposed a search pruning strategy to judge if the candidate interval is within the coverage of each sensor in sensor set generated in *Phase I*, which avoids some unnecessary traversal.

#### 5.2.2. Algorithm

The Algorithm 5 gives the pseudocode for this phase. Firstly, initialize a variable flag to 0 and initialize *TrueSensorSet*. Then, traverse each sensor in *Sensor Set*, for each sensor *s* in *Sensor Set*, set variable flag to the Index of the sensor, for each i from flag to the *Intervals*’ size of sensor s. Assign sensor’s ith interval to *sensorInterval*. If the *sensorInterval* contains start, make a further judgment if it contains end, if so, add sensor s to *TrueSensorSet* and update sensor’s index to flag, break the loop. Otherwise, auto-increment flag, if the start of *sensorInterval* is bigger than the start time of candidate interval, break the loop. Finally, return the *TrueSensorSet*.

#### 5.2.3. Example

**Figure 7:**
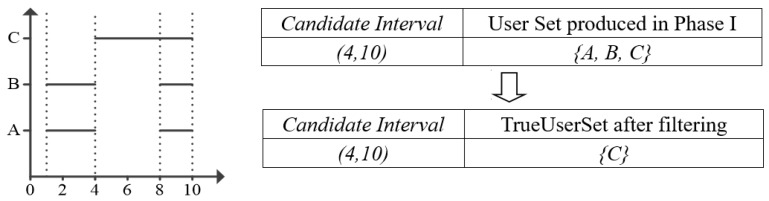
Example in Phase II.

Take the Figure 7 for example, it gives the *Candidate Interval* and its corresponding *Sensor Set* produced in *Phase I*. Assume the *Candidate Interval* is (4, 10), then we traverse all sensors in *Sensor Set*, judge if there exists an interval in each sensor that can cover (4, 10). If so, add this sensor to *TrueSensorSet*. We can find that only sensor C can satisfy the condition.
**Algorithm 5:**Phase II: Filter Fake Sensor.**Input:***Sensor Set,* the set of sensors generated in *Phase I*    *Candidate Interval* generated in *Phase I***Output:**The sensor set after filtering fake sensor1.flag←0; Initialize *TrueSensorSet* to an empty set;2.**for** each *sensor s* in *Sensor Set*3. flag←*s.Index*4. **for**
*i ← flag* to *s.Intervals.size*5.  sensorInterval ← *s.Intervals*.get(i)6.  **if** sensorInterval contains start of candidate interval7.    **if** sensorInterval contains end8.      add s to *TrueSensorSet*9.      s.index←flag10.      break11.    **else**12.      s.index←flag13.      break14.  **else**15.    ++flag16.    **if** sensorInterval.start ≥ start of candidate interval17.      break18.return *TrueSensorSet*

### 5.3. Phase III: Adjust the Candidate Interval

The input of this phase is the candidate interval and its *TrueSensorSet* generated in *Phase II*. While the output is the *trueMap* that contains each candidate interval with its corresponding unique *TrueSensorSet*.

#### 5.3.1. Main Idea

The main idea of this phase is to adjust the candidate interval who has the same *TrueSensorSet* so that each unique *TrueSensorSet* has a unique corresponding candidate interval. The main reason for this is that there exists a situation that several overlapping candidate intervals may have the same *TrueSensorSet*, which will enlarge the final query result and fail to get the accurate maximum range counting sensors and its corresponding optimal interval.

#### 5.3.2. Algorithm

Algorithm 6 gives the pseudocode for this phase. Firstly, initialize a tempSet for storing temporary *TruSensorSet*. Then, for each *Candidate Interval* generated in *Phase I*. If *tempSet* contains no *TrueSensorSet*, add the *TrueSensorSet* into *tempSet* and Store the *Candidate Interval* and its *TrueSensorSet* into *TrueMap*. Otherwise, make a judgement if *tempSet* contains *TrueSensorSet*. If so, continue the loop. Otherwise, add the *TrueSensorSet* into *tempSet* and Store the *Candidate Interval* and its *TrueSensorSet* into *TrueMap*.

#### 5.3.3. Example

**Figure 8:**
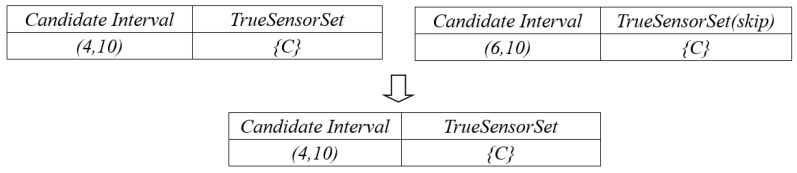
Example in Phase III.

From the example in Figure 8, we can find that candidate interval (4, 10) and (6, 10) both has the same *TrueSensorSet* {C}. Because candidate interval (6, 10) is generated after (4, 10). We can find that *tempSet* contains *TrueSensorSet* {C}. So, we continue the loop without adding candidate interval (6, 10) and its *TrueSensorSet*. Finally, we select the maximum *TrueSensorSet* with its corresponding optimal interval and output the these *SensorIDs.*
**Algorithm 6:****Phase III: Adjust the Candidate Interval.****Input:***Candidate Interval* Generated in *Phase II*    *TrueSensorSet* Genernated in *Phase II***Output:**The *TrueMap* that Stores Each Unique *Candidate Interval* and Its Corresponding *TrueSensorSet*1.Initialize a tempSet;2.**for** each Candidate Interval genernated in Phase I3.  **if** tempSet is empty4.    Add the TrueSensorSet into tempSet5.    Store the Candidate Interval and its TrueSensorSet into TrueMap6.  **else**7.    **if** tempSet contains TrueSensorSet8.        continue9.    **else**10.      Add the TrueSensorSet into tempSet11.       Store the Candidate Interval and its TrueSensorSet into TrueMap

## 6. Experimental Evaluation

In this section, we will discuss the experimental evaluation of BMRC based on the simulation of temporal sensor data set in a sensor monitoring network. Firstly, we will validate the temporal data updating of events’ performance including insert and delete operations in the sensor monitoring network. Then we will compare the conventional baseline approach with BMRC to investigate the performance of maximum range counting query depending on several influencing factors, such as data volumes, time window and time range. All experiments are conducted on a server machine equipped with a 2.70 GHz dual-core Intel Core i5 processor and 8 GB RAM and a 128 GB PCle-based SSD running MacOS Sierra 10.12.1.

### 6.1. Index Performance

**Figure 9:**
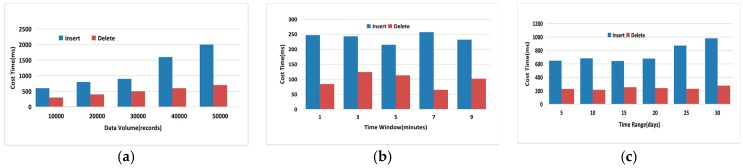
Comparison study for Updating: (**a**) Varying Data Volume; (**b**) Varying Time Window; (**c**) Varying Time Range.

Figure 9 represents the running time of executing an updating affair like insert or delete operation in BMRC in different conditions of data volume, time window and time range. In Figure 9a, we vary the data volume from 10,000 records to 50,000 records, while fixing the duration of time window to 1 min and the time range from 1 July 2013 to 30 July 2013. As we can see from the figure the cost time of both insert and deletion operations is increasing. Moreover, the deletion speed is faster than the insertion one. The time consumptions of these two operations are both within the acceptable time for temporal data updating in real time.

In Figure 9b, we vary the time window from 1 min to 9 min, while fixing the data volume to 10,000 records and the time range for both is 30 days, namely, 1 July 2013 to 30 July 2013. We can find that as the time window grows, both of the insertion and deletion performance is less sensitive to meeting the increasing time window because the time window is just used to filter out the intervals from *Sensor Table* that cannot satisfy the length of time window. As the time window grows, the valid intervals longer than the time window will decrease. However, the obvious randomness comes from the bitmap index adding and deleting bit process. The process of the insertion and deletion algorithm reflects flexibility over the updating events. If the interval to update is too long or too short, it will influence the process of bit changing in *BitPosition*, which will show randomness.

In Figure 9c, we vary the time range from 5 days to 30 days, e.g., 1 July 2013 to 5 July 2013 is a 5 days range, while fixing the duration of the time window to 1 min and the data volume to 50,000 records. The processing time of both operations is also insensitive to the increase of time range of the temporal data. This is because the data volume is fixed to a given value although the time range is growing. The performance of updating affairs is mainly effected by data volume, so the result in Figure 9c shows stability.

### 6.2. Query Performance

**Figure 10:**
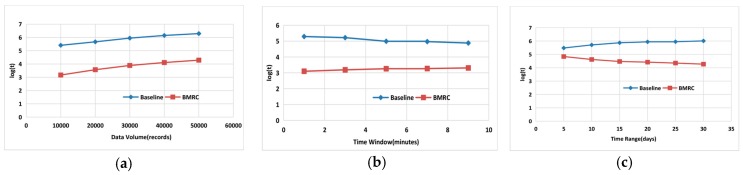
Comparison study for BMRC: (**a**) Varying Data Volume; (**b**) Varying Time Window; (**c**) Varying Time Range.

This section studies the average response time of BMRC. In Figure 10, we compare BMRC with the baseline algorithm for the following three alternatives: (1) Data volume, i.e., the amount of temporal intervals recorded in the sensor monitoring network; (2) Time Window, i.e., a time constraint supposed by a certain query request that the optimal interval must satisfy; (3) Time Range, i.e., the temporal horizon of records monitored in the sensor monitoring network. In order to avoid skewness towards large values of long querying time, all experiments in Figure 10 are plotted with a logarithmic scale of base 10.

In Figure 10a, we vary the data scale of sensor data from 10,000 to 50,000, while fixing the duration of time window to 1 min and the time range from 1 July 2013 to 30 July 2013. As we can see from the performance comparison, the above two approaches follow the same trends. As data volumes grow, the query request execution time of both approaches is also increasing. However, the query time of BMRC is about two orders of magnitude faster than the baseline algorithm. This is because the baseline algorithm need to traverse every step length of the timeline, e.g., from 12:11:00 to 12:12:00, there will be 60 incidences of traversal of the judgment of coverage, whereas the outermost loop in BMRC just needs to traverse all the start times in the *Sensor Table*. Moreover, as the data volumes increase, the sensor number will increase in the *Sensor Table* resulting in the increase of time number, which both prolong the traversal time consumption in the above two algorithms.

In Figure 10b, we vary the time window from 1 min to 9 min, while fixing the data volume to 10,000 records and the time range is both 30 days, namely, from 1 July 2013 to 30 July 2013. With the increase of the duration of the time window, the query execution time of the above approaches tend to become stable. However, the query time of BMRC approach is nearly two orders of magnitude faster than the baseline algorithm because the time window is just used to filter out the intervals from *Sensor Table* that cannot satisfy the length of time window constraint. As the time window grows, the valid intervals longer than the time window will decrease, however, the number of all the valid times in the *Sensor Table* seems to be unchanged, so the outermost loop times in BMRC will remain stable.

In Figure 10c, we vary the time range from 5 days to 30 days, e.g., 1 July 2013 to 5 July 2013 is a 5 days range, while fixing the duration of the time window to 1 min and the data volume to 50,000 records. With the increase of the time range of the temporal data, the query execution time of the baseline approach is increasing gradually, but the time consumption of BMRC is decreasing gradually. Anyhow, the BMRC approach is nearly two orders of magnitude faster than the baseline algorithm because the fixing of data volume and the increasing of time range will influence the data sparsity. Intervals among small time range will be more intensive than a large time range. This will lead to the significant influence in *Phase II* (*Filter Fake Sensor*). For example, if there are 50,000 records, each sensor among the 5 days range will have more records than that in the 25 days range. For a 5 days range, *Phase I* (*Get Maximum Sensor Set*) will likely produce a larger maximum sensor set that will slow down the process in *Phase II* (*Filter Fake Sensor*). However, the larger time range will just increase the number of traverse times for baseline approach.

### 6.3. Inside Maximum Range Counting Query

This section discusses the internals of maximum range counting query algorithm in terms of the performance and average ratio of each phase separately.

**Figure 11:**
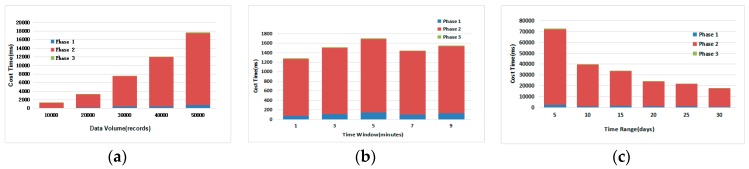
Cost Time for Different Phases in Query Process: (**a**) Varying Data Volume; (**b**) Varying Time Window; (**c**) Varying Time Range.

(1) Time consumption in Each Phase:

From the experiment results in the three subfigures in Figure 11, we can draw a conclusion that Phase II takes has the largest time consumption compared to the other two phases, while Phase I takes the second place and Phase III takes the least time.

In Figure 11a, we vary the data volume from 10,000 records to 50,000 records, while fixing the the duration of the time window to 1 min and the time range from 1 July 2013 to 30 July 2013. As we can see from the figure the cost time of a query is increasing with the increase of data volume. Moreover, the time consumption can be few seconds when the data volume is small. *Phase I* is also increasing gradually because the number of sensors and time in *Sensor Table* is increasing. Meanwhile, *Phase III* remains stable.

In Figure 11b, we vary the time window from 1 min to 9 min, while fixing the data volume to 10,000 records and the time range is both 30 days, namely, 1 July 2013 to 30 July 2013. We can find that as the time window grows, the query time consumption is gradually decreasing. However, when the time window is set to 3 min, the query time increases sharply, because the query results contain several optimal intervals that have the same maximum sensor set, which will prolong the processing time of the filter fake sensor procedure. The number of all the valid times in *Sensor Table* however seems to be unchanged, hence, the outermost loop times in BMRC will remain stable, which will keep the total time consumption stable. The other phases almost remain in the same status, which indicates they are insensitive to the time window factor.

In Figure 11c, we vary the time range from 5 days to 30 days. e.g., 1 July 2013 to 5 July 2013 is a 5 days range, while fixing the duration of the time window to 1 min and the data volume to 50,000 records. The query time is sensitive to the increase of time range of the temporal data. As the time range increases, the time consumption is decreasing gradually because when the data volume is fixed, the smaller the time range, the bigger the number of *Intervals* per unit sensor will be, which will cause more time consumption during *Phase II*.

**Figure 12:**
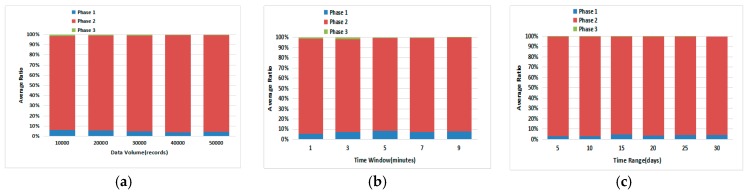
Average Ratio for Different Phases in the Query Process: (**a**) Varying Data Volume; (**b**) Varying Time Window; (**c**) Varying Time Range.

(2) Average Ratio of Each Phase:

From the four figures in Figure 12, we can see that *Phase II* is the largest component of the query process, while *Phase I* takes the second place and *Phase III* takes the least time. Overall, the three phases in Figure 11 and Figure 12 indicate the value and necessity to take them working together for the performance of query process in BMRC.

## 7. Conclusions

In this paper, we propose BMRC, a bitmap index structure-based maximum range counting approach for processing temporal sensor data dynamically in sensor monitoring networks. The temporal data monitored by each sensor is collected for the optimal interval query request which results in the maximum number of sensors that monitor the same event. Firstly, BMRC obtains the temporal sensor data to construct a data structure for query preparation. Based on the data structure, BMRC utilizes a bitmap encoding strategy to encode every unique time with its corresponding bitmap, which can support temporal data updating events in real time. Extensive experiments indicate that BMRC can respond to query requests in only a few seconds for tens of thousands of temporal data, which proves its performance and scalability.
